# Rationale, design and methods of the Study of Work and Pain (SWAP): a cluster randomised controlled trial testing the addition of a vocational advice service to best current primary care for patients with musculoskeletal pain (ISRCTN 52269669)

**DOI:** 10.1186/1471-2474-15-232

**Published:** 2014-07-10

**Authors:** Annette Bishop, Gwenllian Wynne-Jones, Sarah A Lawton, Danielle van der Windt, Chris Main, Gail Sowden, A Kim Burton, Martyn Lewis, Sue Jowett, Tom Sanders, Elaine M Hay, Nadine E Foster

**Affiliations:** 1 Research Institute for Primary Care & Health Sciences, Keele University, Keele, Staffordshire ST5 5BG, UK; 2 Institute for Research in Citizenship and Applied Human Sciences, University of Huddersfield, Huddersfield, UK; 3 School of Health and Population Sciences, University of Birmingham, Birmingham, UK

**Keywords:** Cluster randomised trial, Musculoskeletal pain, Primary care, Vocational advice, Case management, Work

## Abstract

**Background:**

Musculoskeletal pain is a major contributor to short and long term work absence. Patients seek care from their general practitioner (GP) and yet GPs often feel ill-equipped to deal with work issues. Providing a vocational case management service in primary care, to support patients with musculoskeletal problems to remain at or return to work, is one potential solution but requires robust evaluation to test clinical and cost-effectiveness.

**Methods/Design:**

This protocol describes a cluster randomised controlled trial, with linked qualitative interviews, to investigate the effect of introducing a vocational advice service into general practice, to provide a structured approach to managing work related issues in primary care patients with musculoskeletal pain who are absent from work or struggling to remain in work. General practices (n = 6) will be randomised to offer best current care or best current care plus a vocational advice service. Adults of working age who are absent from or struggling to remain in work due to a musculoskeletal pain problem will be invited to participate and 330 participants will be recruited. Data collection will be through patient completed questionnaires at baseline, 4 and 12 months. The primary outcome is self-reported work absence at 4 months. Incremental cost-utility analysis will be undertaken to calculate the cost per additional QALY gained and incremental net benefits. A linked interview study will explore the experiences of the vocational advice service from the perspectives of GPs, nurse practitioners (NPs), patients and vocational advisors.

**Discussion:**

This paper presents the rationale, design, and methods of the Study of Work And Pain (SWAP) trial. The results of this trial will provide evidence to inform primary care practice and guide the development of services to provide support for musculoskeletal pain patients with work-related issues.

**Trial registration:**

Current Controlled Trials ISRCTN52269669.

## Background

Musculoskeletal pain and in particular acute back pain are major contributors to short term (less than 20 working days) and long term (greater than 20 working days) work absence, accounting for 38% and 37% of short-term absence respectively in manual jobs and 37% and 28% respectively in non-manual jobs [1]. However, around one third of all work absence is attributable to long-term musculoskeletal conditions accounting for long-term absence in 37% of manual and 34% of non-manual jobs [1].

### Current policy regarding health and work

The health service costs and lost capacity in the workplace have made health and work a key target for public policy [2]. In the UK the Government is actively aiming to reduce the number of employees signed off sick each year [3]. Provision of occupational health in the workplace in the UK is currently limited. Even when occupational health services are broadly defined, only 15% of UK employers provide such a service and these are generally the larger organisations [4]. Occupational health services are even less likely to be provided in Small and Medium Enterprises (SMEs), which employ an estimated 13.5 million people [4,5]. For the vast majority of SME employees, in the UK and elsewhere, the first line of occupational health care is their primary care practitioner and there is a strong case for primary care services being involved in work-related health interventions by providing more options to refer patients [6].

### Limitations of current occupational health care for musculoskeletal pain

The benefits of remaining active despite pain have been well documented in workers with musculoskeletal pain and back pain in particular, leading to less sick leave, less time on modified duties and a reduction in pain recurrence [7-10]. A review of vocational rehabilitation highlighted primary care as a key arena in which to address the issue of work with patients [11]. Although there are guidelines in place to support primary care practitioners in providing appropriate advice about work [12-17], many GPs have limited training in work issues [18] and they often report that they feel ill-equipped to deal with patients’ concerns about work [19,20]. In the UK this is particularly important given the introduction of the ‘Statement of Fitness for Work’ which replaces the sickness certificate, requiring GPs to assess fitness for work and provide their patients with more specific advice regarding activities (e.g. altered hours or modified activities) that may facilitate successful return to work.

### Interventions to facilitate return to work

Initiatives addressing health and work have been predominantly policy driven, such as Job Centre Plus, the Job Retention and Rehabilitation Pilot and the Pathways to Work initiatives in the UK [21,22] and are often directed towards people who have extended work absence (greater than 6 months). Yet evidence from back pain research suggests that the longer an individual is out of work, the harder it is for them to get back into work [23], therefore it is logical to tackle absence before it becomes long-term. Evidence suggests that intervening in the early stages of sickness absence may be effective for many people with musculoskeletal conditions and yet most initiatives currently are directed towards longer-term absence from work [6,9,11].

In the research arena there are a range of interventions addressing shorter term absence that have been tested to examine their effects on work absence, these include but are not limited to back schools, exercise programmes, work hardening programmes and educational programmes [24,25]. However, these interventions have mostly been undertaken in the workplace, and they have been tailored to the specific needs of the organisations in which they have taken place.

There are methods by which the impact of health on work may be addressed on an individual level, rather than a policy level or organisational level, to ensure that patients receive support in managing their health in the context of their work. In Denmark a multidisciplinary intervention including case management has been evaluated in the rehabilitation of employees sick-listed for 4–12 weeks due to low back pain [25] and “Fit for Work” services, based on case managed, multidisciplinary approaches providing treatment, advice and guidance for people in the early stages of sickness absence have been recommended in the UK [6]. Case management can be defined as a “goal oriented approach to keeping employees at work and facilitating an early return to work” [26]. Given that early intervention is advocated, that musculoskeletal conditions are a common cause of work absence and that many individuals seek their healthcare initially from their primary care practitioner, testing a service located in primary care that can address work issues early on in patients with musculoskeletal conditions is appropriate. However, such a service needs to have a broad enough scope to ensure appropriate advice for the majority of patients whilst still providing a tailored service, therefore the case management approach is the most appropriate model.

## Aim

This paper describes the rationale, design and methods for a cluster randomised controlled trial and linked qualitative interviews, to investigate the clinical and cost-effectiveness of introducing a vocational advice service into general practice, with the aim of providing a structured approach to managing work related issues for primary care patients with musculoskeletal pain who are absent from work or struggling to remain in work.

The principal research question of the SWAP trial is:

What is the effect of the addition of a vocational advice service to best current care compared to best current care alone, for adults with musculoskeletal conditions in primary care absent from or struggling to remain in work? The secondary questions are:

1. Is the addition of a vocational advice service to best current care compared to best current care alone for adults with musculoskeletal conditions in primary care absent from or struggling to remain in work cost effective?

2. What are the experiences of patients, GPs/NPs and vocational advisors of a primary care based vocational advice service?

## Ethical approval

Ethical approval was obtained from NRES Committee West Midlands – Staffordshire in April 2012 (REC reference: 12/WM/0020).

## Methods

### Trial design

SWAP is a pragmatic cluster randomised controlled trial with two parallel arms and incorporates economic evaluation and linked qualitative interviews. The unit of randomisation is the general practice with data collected from individual participants.

### Settings and clusters

This cluster trial will take place in six general practices in the South Staffordshire area of the Staffordshire and Stoke-on-Trent Partnership NHS Trust in the UK. Informed consent for practices to participate will be provided by the senior GP partner. Patients will follow the care to which their practice is randomised with identical participant information for both arms explaining that their local musculoskeletal services are being evaluated using patient self-complete questionnaires and medical record review. A second information sheet was used to inform participants about the interview study. Individual patients will be able to opt-out of the questionnaire data collection and the interview study.

### Randomisation and allocation concealment

GP practices are the unit of randomisation. Practices recruited to the cluster trial will be matched based on list size, with matched practices subsequently randomly allocated to the intervention or control arms. Allocation concealment for participating GPs and vocational advisors is not possible but individual participants will not know the allocation of their practice. In this cluster RCT individual participants will not know they are in a trial as the patient information will not mention randomisation of practices and will simply inform participants that local musculoskeletal services are being evaluated. In addition, data entry staff who input data from study questionnaires will be blind to allocation. Analysis of the primary outcome will be carried out by two statisticians (one of which will be blinded to treatment arm). The results will be reviewed and agreed by both statisticians, with one statistician remaining blind until agreement on final estimates is reached.

### Participant eligibility criteria

Adults aged 18 to 70 years consulting in primary care with musculoskeletal pain will be eligible to take part if they are:

● Currently employed (paid)

● Current sickness absence of less than 6 months duration (either GP or self-certified absence) due to musculoskeletal pain OR

● Patients considered by the GP (or a nurse practitioner (NP)), during the consultation, to be struggling with work due to musculoskeletal pain

Exclusion criteria are:

● Patients with symptoms indicative of possible serious pathology, requiring urgent medical attention

● Patients unable to read and speak English

● Patients with serious mental health problems who are vulnerable and for whom participation in the study would be detrimental (at the GP’s discretion)

● Those who have long term work absence (greater than 6 months)

● Pregnancy or those patients on maternity leave

### Participant recruitment

Potential participants will be identified when they consult their GP practice with musculoskeletal pain. When a Read code for a musculoskeletal pain problem is entered in the electronic medical record, a computer template will be activated. The template will prompt the GP or NP to record whether the patient is struggling to remain in work or absent from work. Patients who are present when the GP or NP completes the computerised template and express an interest in the research will be given a SWAP information pack at the GP practice. The records of patients who are not present with the GP or NP when the computer template is completed, will be ‘tagged’ and downloaded on a weekly basis. The local NIHR Clinical Research Network Primary Care administrator will post an information pack to these patients.

**Figure 1:**
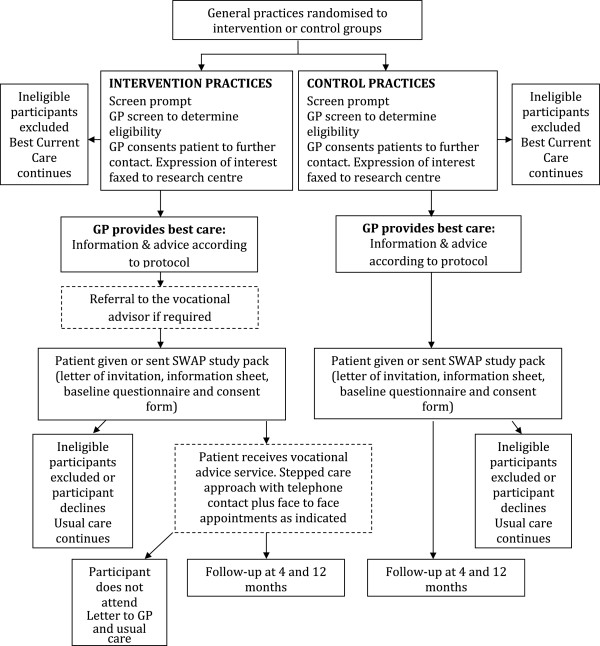
SWAP trial flowchart.

Electronic templates have been successfully implemented in previous studies carried out by the Arthritis Research UK Primary Care Centre at Keele University and are now routinely used to identify participants for research studies based in general practice [27]. The information pack will include a letter of invitation, a participant information sheet, consent form, self-completion questionnaire (baseline data collection) and a pre-paid reply envelope. The letter of invitation will invite potential participants to take part by completing a consent form and returning the baseline questionnaire. The information sheet will provide further details about the trial. As participants will not be individually consented to randomisation, participants in both arms of the trial will be asked to give written consent to take part in a study investigating work related musculoskeletal problems and local health services by completing three questionnaires (at baseline, 4 months and 12 months) and to allow the research team access to their medical records to identify GP certified Fit Notes in the 6 months prior to consent and during the follow-up period, and to review further health care utilisation for cost analysis. The same procedure will be followed for both the intervention and control practices. A flowchart illustrating the SWAP trial is shown in Figure 1.

### Description of intervention and control arms

All GPs and NPs working in both the intervention and control practices will be invited to participate in an evidence update session discussing best current care for the management of musculoskeletal pain and work. This aims to ensure that all patients receive the same level of best current care, allowing the added benefit of the vocational advice service to be assessed. The evidence update session will centre on providing GPs and NPs with information to ensure that the correct advice is provided to patients about working with musculoskeletal pain. It will focus on the key messages that a) work is usually good for people with musculoskeletal pain, b) long periods of absence from work are harmful, c) musculoskeletal pain can often be accommodated at work with appropriate adjustments and support, if necessary d) planning and supporting return to work are important parts of clinical management. In addition to these key messages GPs and NPs will be provided with advice about how to approach discussing difficult issues with patients such as negotiating absence or modified duties in the workplace.

#### Control practices

Control practices will provide best current care by GPs and NPs in addition to all other usual care that patients may require for their musculoskeletal pain.

#### Intervention practices

**Figure 2:**
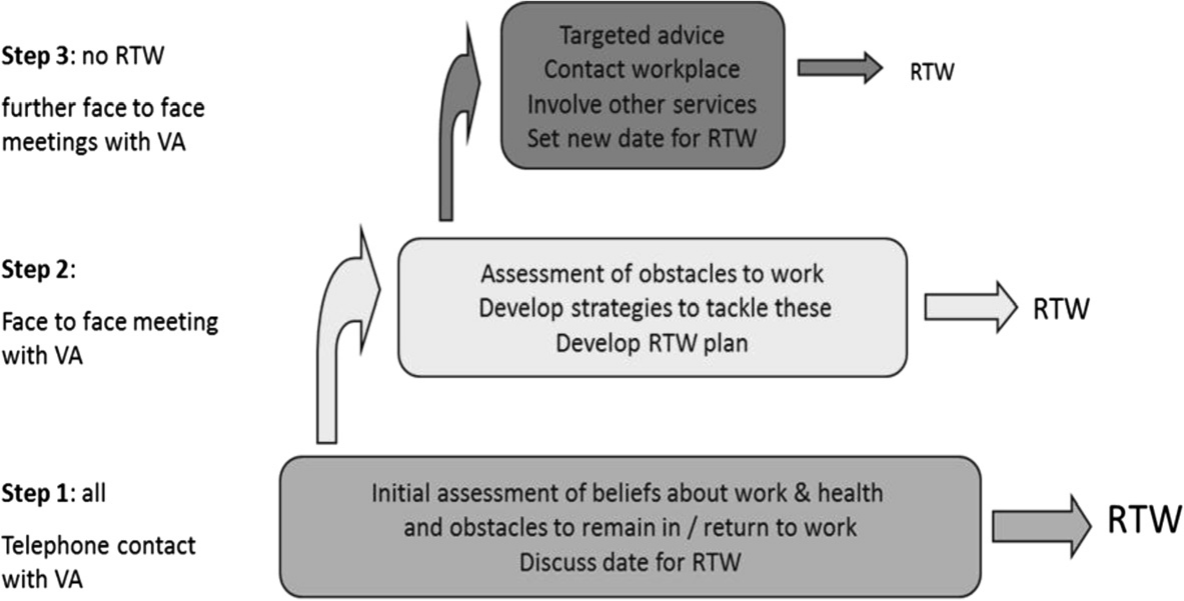
Model of stepped care provided by the vocational advisor (VA) in addressing return-to-work (RTW).

Intervention practices will also provide best current care and all other care as usual. In addition to best current care a vocational advice service will be available to intervention practices. Patients who require help and support in remaining at or returning to work may be referred to the vocational advice service by their GP or NP, irrespective of whether they also consent to participate in the research evaluation. Patients who are referred to the vocational advice service will be contacted by a vocational advisor, seven days after receipt of the referral who will help the patient to identify and overcome obstacles to remaining at or returning to work. The vocational advisor will wait for seven days before contacting the patient to minimise the number of participants who provide baseline data after having contact with the vocational advisor. It is expected that obstacles to return to work or remaining at work will fall into several categories, and the Flags model of management [28] of the health and work interface will be used to structure the vocational advice service. The Flags model focuses on the identification of obstacles to working with health conditions, development of a plan to manage health and work, taking action to address the issues each individual patient is facing with respect to managing their musculoskeletal condition in the workplace and re-evaluating the patient’s situation regularly until a sustained return to work is achieved [28]. The model is a “light touch” approach based around the principles of case management and stepped care, with vocational advisors providing a goal oriented approach to return to work or remaining in work and with patients being able to “step up” the support they receive when necessary (Figure 2). Stepped care has been used successfully in the management of mental health conditions and has begun to be used successfully in pain management [29,30]. Patients will be eligible for continued vocational advice until they have a sustained return to work, feel able to manage their health condition in the context of their work, or until they have been absent from the workplace for a total of six months, at which point they will be directed towards other appropriate services.

### Audit of intervention

The vocational advisors will complete case report forms for each participant in the intervention arm, recording basic demographic details, assessment findings and their management plan, and the type and number of contacts each participant has with the vocational advisor. An audit on the completion of the case report forms against the vocational advisor clinical case notes will assess whether patient demographics, contacts with the vocational advisor, details of the assessment and management plan, and any contact with other stakeholders (e.g. healthcare providers, employers) are consistently and accurately recorded on the case report forms.

### Training and mentoring of vocational advisors

Four health care practitioners have been recruited to vocational advisor posts for the trial. They attended a four day training programme on managing work issues within primary care for patients with musculoskeletal conditions. The training programme was based on stepped care and case management principles. The vocational advisors also attended a half day update just prior to the start of the vocational advice service. Monthly mentoring meetings will be scheduled throughout the study where the vocational advisors have the opportunity to request further clarification on any aspect of the teaching, and discuss individual cases both with colleagues and the trainers (a consultant physiotherapist and clinical psychologist who are experienced in managing work related issues).

### Sample size

In summary, 330 recruited participants (165 per arm) in the SWAP trial will give 80% power to detect at least a mean difference of 10 days (days off work between baseline and 4 months) given an expected standard deviation of 25 [31], and 5% two-tailed significance level. The primary analysis method is described below and does not directly involve computing mean difference (but incidence rate ratio via a Poisson/negative binomial process). However, the above calculation holds when applying Normal approximation to the binomial distribution as is generally accepted when the combination of rate of occurrence and sample size is sufficiently large (i.e. both np and n(1-p) exceed 5, where p in this case denotes the probability of taking time off work in any given day, and generally for any Poisson process where the mean/rate is 10 or greater). The model proposed for the analysis of the primary endpoint (number of days off work) is more suited for analysing discrete data than general linear models which are suited to continuous data.

The total sample size requirement for analysis of an un-clustered unadjusted analysis based on detecting a mean difference of 10 with 80% power and two-tailed 5% significance level (assuming an SD of 25) is 200 individuals (100 per study arm). The above calculated total sample size requirement of 330 participants for the SWAP trial takes into account three levels of inflation (of the unadjusted figure): (i) 20% (×1.2 magnification) through clustering of data (at practitioner- level) based on an ICC for between-practitioner effects of 0.05 [32] with anticipated average cluster size of 5 per practitioner; (ii) 15% owing to variation in expected recruitment rates between GPs (based on an expected coefficient of variation of 0.65) [33], and (iii) 20% allowance for loss to follow-up at 4 months.

### Participant (baseline) characteristics

Baseline data by trial arm will be summarised and presented. Baseline characteristics are to be compared between arms, and presented at the level of: (i) GP practice clusters, and (ii) Patient characteristics.

Baseline data for GP practice characteristics include data on the stratified variable for randomisation – i.e. practice list size. Also, number of GP practitioners, median index level of deprivation for the practice, mean age, and gender (male/female) distribution of practice populations will be described.

Also, comparison will be made between participants’ demographic, pain/disability and quality of life characteristics. Mean (SD) and median (IQR) will be applied to normal and skewed numerical data respectively. Frequency counts and percentages will be presented for nominal and ordered data.

Balance of baseline characteristics is particularly important to establish for cluster trials given the (higher level) unit of randomisation. A lack of balance is indicative of differential selection of patients to the trial across the treatment arms (though appreciating that random difference will occur due to randomisation and between-practice variations). A further limitation of the design is that ‘baseline’ assessment occurs after initial GP or NP consultation and therefore a difference in pain management responses may occur within that period – i.e. prior to baseline assessment (so baseline, defined as the date the participant completes the first questionnaire, in this context is not a true ‘baseline’ of where baseline is usually considered to be, prior to the start of treatment). We may expect differences in treatment (such as issuing of sickness certificates) to occur by this baseline assessment and in particular there may already be differences in approach that may influence the primary outcome by the time of this first self-report baseline assessment. Therefore, the primary analysis will not adjust for baseline pain intensity (though this adjustment will be carried out as a sensitivity analysis - see Analysis section for further details). No formal statistical testing will be carried out for differences in baseline characteristics as this is not an ‘outcome’ for the trial.

#### Assessment of potential bias

*Selection bias*: Over the period of recruitment the number of patients who consult with musculoskeletal pain and are potentially eligible for the trial as coded by the GP or NP on the computer prompt will be recorded in the intervention and control practices. Any evidence of selection bias in rate of uptake to the research and in baseline descriptive statistics between the control and intervention practices will be explored. Demographic comparisons will be drawn between trial participants, non-participants and screened patients who do not take part.

*Attrition bias*: Differences between individuals that are followed up and those who dropout is a concern, and may result in between-arm bias in estimates particularly if dropout is unequal between the two trial arms and analysis fails to take into account appropriate adjustment for missing data. Thus, we will compare: (i) baseline characteristics of those who are successfully followed up at 4 months against those who dropout to assess whether those missing are related to observed baseline factors, (ii) attrition rate between trial arms to assess whether there is a differential dropout rate. Statistical adjustment will be carried out to help address issues of imbalance in characteristics.

### Outcome assessment

#### Primary outcome measure

The primary outcome measure is number of days off work over 4 months from entry into the trial. This is based on response to the following questions in the 4-month self-report questionnaires: “Have you **taken time off work** during the **last 4 months** (since your last questionnaire) because of your pain? **If yes**, please write in the **number** of days, weeks or months you were off work due to your pain in the **last 4 months.** (i.e. between baseline and 4 month follow up assessments)”. Days off work in this context jointly captures sick leave issued by the GP and shorter length self-certified absences that don’t require GP sign-off.

#### Secondary outcome measures

Self-reported time off work (in binary form (yes/no)) will be a secondary outcome. We will also undertake a separate analysis to compare the proportion of participants in the two trial arms that are issued a GP sickness certificate in the first 4 months (through review of medical records for those who provide consent to medical record review). Secondary evaluation will also look at self-reported time off work and medical record review based sick certification periods over 12 months follow-up.

Other secondary outcome measures include the Self-efficacy to Return to Work Questionnaire [34], pain intensity (0–10 rating scales), bothersomeness (1–5 rating scale), global assessment of change and work performance (SPS6).

Table 1 summarises the outcome measures and their respective time-points of data collection.

**Table 1:** Outcome measures and timing of data collection

**Measures**	**Description**			
		**Baseline**	**4 months**	**12 months**
Primary outcome measures				
Absence	Work absence self-reported and GP certified including duration of absence, and struggling at work	✓	✓	✓
Secondary outcome measures				
Pain intensity	Three questions: 0–10 scales for ‘present’, ‘usual’ and ‘least’ pain in last 2 weeks	✓	✓	✓
Bothersomeness	Single question: 1–5 point scale	✓	✓	✓
Change	Global Assessment of Change – one question	✗	✓	✓
Return to work self-efficacy	Self-Efficacy Return to Work Questionnaire	✓	✓	✓
Work performance	Stanford presenteeism scale 6 (SPS6), plus single question on performance at work	✓	✓	✓
Prognostic indicators or potential mediators				
Demographics	Gender, date of birth, socio-economic status (recent paid job title)	✓	✗	✓
Employment	Current work situation	✓	✗	✓
Episode duration	One question on duration of current episode, plus one question on time since pain-free month	✓	✗	✗
Pain elsewhere	Additional pain locations indicated on a Body Manikin	✓	✗	✗
Illness perceptions	Musculoskeletal Illness Perceptions Questionnaire Revised (IPQ-R) Short-Form	✓	✓	✓
Symptoms of anxiety and depression	Hospital Anxiety and Depression Questionnaire (HADs)	✓	✓	✓
Pain self- efficacy	Pain Self Efficacy Questionnaire (PSEQ)	✓	✓	✓
Attitudes & beliefs (patients) re. work & health	Newly developed questionnaire	✓	✓	✓
Content of GP/NP consultation	Questions regarding topics covered by the GP/NP (including work)	✓	✗	✓
Treatment satisfaction	Question regarding satisfaction with treatment	✗	✓	✗
Health economic measures				
Health care utilisation	Health Care Utilisation Questions	✗	✗	✓

### Analysis

Data will be analysed after the 4 month follow-up and the 12 month analysis, which will include Health Economic data, will then follow. For the primary analysis (time (days) off work in the first 4 months), the proposed analysis is by hierarchical negative binomial regression adjusting for age, gender, and GP practice size (at the GP-cluster level). Multi-level robust Poisson and zero-inflated models taking into account clustering of data and over-dispersion in the spread of data will also be scrutinised (alongside the negative binomial model). The goodness of fit of each model for observed versus predicted values will be scrutinised (comparisons will be drawn through a likelihood-ratio test and Akaike’s Information Criterion (AIC)). Mixed-models (linear- or generalised- as appropriate to numerical and categorical outcome data, respectively) will be fitted to estimate and test for between arm effects across primary and secondary outcome measures – adjusting for baseline covariates (as indicated above). An intention-to-treat approach analysing participants as per randomised allocation will be followed.

A limitation to the design/methods of this trial is the small number of GP practice clusters (i.e. units of randomisation). It has been reported that a minimum number of clusters for a valid methodological evaluation is four per arm (our trial has three GP practices per arm) [35]. Much of this concern centres on the assumptions for the hierarchical model, and the fact that any cluster level analysis (only) will fail to detect a statistically significant p-value (at the level of the customary 5% two tail testing). Hence, we propose to carry out the hierarchical model with individual practitioners (GP/NPs as opposed to GP practice) as the upper-level random factor. GP/NPs are likely to be the main contributors to the variation in sickness certification between GP practices and may therefore be considered to be reasonable substitutes [36].

Descriptive statistics on numbers of participants and proportion of participants who take time off work in each arm will be reported for the primary outcome. The adjusted effect estimate (incidence rate ratio), 95% confidence interval and p-value for the test of association for the primary measure will be presented. Similarly, mean scores (SDs) for numerical outcomes and frequency counts and percentages for categorical data will be presented for secondary outcome measures – as appropriate to the scale of the data. Mean differences and 95% CIs and odds ratios with 95% CIs will be presented for all secondary outcomes – as appropriate to the scale of the data.

#### Sensitivity analysis

The following sensitivity analyses are planned:

1. Evaluation of the primary outcome measure (number of days off work) by robust Poisson and zero-inflated models.

2. The main evaluations will utilise minimal covariate adjustment (owing to the fact that differences may already be inherent in baseline assessment due to the time-scale of return of questionnaires following the initial GP/NP consultation). However, we would not anticipate any real difference in outcomes in such a short time period particularly as the patients in the intervention practices will not have been contacted by a vocational advisor until at least 7 days after receiving the baseline questionnaire. Greater covariate adjustment is also relevant in that it helps safeguard the analysis against major selection bias and/ confounding bias. Thus, as a sensitivity analysis, we will carry out statistical modelling that includes additional baseline adjustment by further including pain intensity, time off work at baseline (at the individual level) as well as corresponding baseline score (if applicable).

3. A second sensitivity analysis will be carried out at the upper cluster level (individual practitioners) using non-parametric sum rank test and permutations test. Individual-level regression methods may not be reliable and the distributional assumptions difficult to verify when the number of units of analysis are small – in such circumstances, as is the case in this trial, it is recommended to carry out a simple crude analysis that is not dependent on distributional assumptions (in this case a simple non parametric comparison since the primary outcome of interest is likely to be highly skewed) [37].

4. Per protocol evaluation (further sensitivity analysis of the primary outcome): A per protocol evaluation will be undertaken comparing the primary outcome for those participants in the intervention practices who engaged with any aspect of the vocational advice service (at least one contact by telephone) versus ‘comparable' participants in the control practices. A complier average causal effect analysis will be performed to provide an unbiased estimate of ‘per protocol’ effect by adjusting the per protocol estimate (on the assumption that a similar level of non-compliance would be expected for the control arm).

Subgroup analyses: Evaluation of the primary outcome measure will be carried out to examine whether time off work/number of days absenteeism is different across different baseline subgroups by: return to work self-efficacy, location of pain (spinal pain versus pain in other areas), and duration of work absence. Statistical estimates will be obtained through including interaction terms in the statistical model of treatment effect.

### Economic evaluation

The economic evaluation conducted alongside the SWAP trial will determine the cost-effectiveness and return on investment of the vocational advice service (cost-benefit analysis) in comparison to best current care.

A cost-consequence analysis will initially be reported, describing all the important results relating to costs and consequences (across the full range of clinical outcomes). Subsequently, two methods of economic evaluation will be used. A cost-effectiveness analysis will be undertaken from a healthcare perspective to determine the cost per additional day of work absence avoided. A cost-benefit analysis will also be undertaken from a broader societal perspective to calculate the net societal benefit of the vocational advice service, by subtracting the difference in direct health care costs (costs) between the groups from the difference in indirect productivity costs (benefits) between the trial arms.

### Costs

Information on time off work will be collected from the postal questionnaires completed by patients at 4 months and 12 months. Health care resource use will be collected in the 12 month questionnaire. Health sector costs will include primary and secondary care contacts, investigations, medication and contacts with other health care professionals such as physiotherapists and occupational therapists (both through the NHS and private). Data on musculoskeletal pain related time off work and health care resource use will also be available from the medical record review. Questions on patients’ personal expenditure will concentrate on private health care use and over-the-counter treatments. Questions on time off work and occupation will provide information required to calculate the indirect (productivity) costs (benefits). In order to obtain the cost of the vocational advice service, information on the type and number of contacts with the vocational advisor (telephone calls or visits) will be obtained and unit costs applied to calculate overall cost of the intervention.

Resource use will be multiplied by unit costs obtained from standard (national) sources and health care providers [38-40]. Due to the lack of nationally representative unit cost estimates for private health care, this care will be costed as the NHS equivalent. Patient reported costs for over-the-counter treatments will be used.

### Health economic outcomes

The outcome measure for the cost-effectiveness analysis is self-reported number of days absent from work. In the cost-benefit analysis, benefits will be estimated from the productivity losses. These will be calculated using data collected on employment status at every time point and number of days off work due to their musculoskeletal pain problem. Information on occupation, further details of typical work activities and the nature of their employment (full time or part time) will be sought in follow-up questionnaires. The average wage for each respondent will be identified using UK Standard Occupational Classification coding and annual earnings data for each job type [41,42]. The analysis will use the human capital approach, and the self-reported days of absence will be multiplied by the respondent-specific wage rate. The human capital approach assumes that the value of lost work is equal to the amount of resources an individual would have been paid to do that work, and values productivity losses as a result of morbidity (or mortality) by measuring time lost from work and multiplying this with the gross wage of the person.

### Cost-consequence, cost-effectiveness and cost-benefit analysis

The health economic analysis will estimate the incremental cost-effectiveness and the cost-benefit of the intervention in comparison with best current care. Costs for the trial arms will be presented for each broad cost category (health care costs, patient-incurred costs, productivity costs) and disaggregated within each of these cost categories. An incremental cost-effectiveness analysis will be conducted from a healthcare perspective using information on time off work to calculate the cost per additional day of work absence avoided. A cost-benefit analysis from a broader societal perspective will calculate the net societal benefit of the intervention in monetary terms, by subtracting the difference in costs from the difference in benefits (productivity losses). Subsequently, a return on investment will be calculated by dividing the net benefits of the vocational advice service (gain minus cost) by the net costs of the intervention. The base-case analyses will use self-reported patient information on health care utilisation over a 12 month period.

The data for costs is likely to have a skewed distribution therefore a non-parametric comparison of means (e.g. bootstrapping) will be undertaken to estimate confidence intervals around costs. Mean substitution techniques (for individual-item missing resource use data) and multiple imputation techniques (resource use data) will be carried out to ensure that all trial participants are included in the final analysis. Clustering of data by GP/NP will be taken into account through a multi-level approach, in line with the main statistical analysis. Adjustment for baseline covariates will focus on the same variables as outlined for the primary clinical evaluation.

The robustness of the base-case results will be explored using sensitivity analysis. This will explore uncertainties in the trial based data itself and the methods employed to collect and analyse the data. An available case analysis will be conducted as an alternative to using a multiple-imputed data set. A further sensitivity analysis will be undertaken using health care resource use data solely obtained from the medical record review. Uncertainty will be explored through the use of cost-effectiveness acceptability curves (CEACs); these plot the probability that the addition of a vocational advice service is cost-effective against threshold values for cost-effectiveness.

### Qualitative research

#### Qualitative methods

In linked qualitative interviews we will explore experiences of the vocational advice service from the perspectives of GPs and NPs in the intervention practices who can refer patients to the service, patients who access the service with work related problems and vocational advisors who are delivering the service. GPs and NPs (up to n = 15) will be interviewed both prior to the start of the new service and 12 months later. Patients (n = 20) who have consented to the research evaluation will be opportunistically invited for interview following discharge from the care of the vocational advisors. Vocational advisors (n = 4) will be interviewed four times, prior to the start of the new service and at 1, 6 and 12 months after the vocational advice service commences. These longitudinal interviews will explore how their knowledge, confidence and experience of providing the vocational advice service evolves over time.

#### Qualitative analysis

Interviews with GPs/NPs, patients and vocational advisors will initially be coded in N-vivo 9 and subsequently analysed in search of common themes and differences, using the constant comparative framework, based on the broad principles of grounded theory [43]. Although each set of interviews will be coded separately (using separate coding frameworks), each dataset will subsequently be analysed as a whole in search of similarities and differences across GPs/NPs, patients and vocational advisors. Samples of early interviews will be independently coded by members of the multidisciplinary research team, coding frameworks agreed and coded data analysed in search of themes at multidisciplinary research analysis meetings. The themes will be analysed through in-depth discussion to examine plausibility and validity to develop a robust thematic framework (or conceptual model); specifically patients’, vocational advisors’ and GPs/NPs’ perceptions of the acceptability, benefits and limitations of the vocational advice service. The constant comparative method will provide a means of identifying similarities and differences in the qualitative data, whilst the longitudinal dimension of the qualitative interviews with VAs (baseline, 1 month, 6 months and 12 months) and GPs/NPs (baseline and 12 months) will identify changes over time in attitudes and experiences towards the acceptability and added value (or otherwise) of the vocational advice service.

##### Trial timeline

Trial recruitment commenced in July 2012. We aim to recruit 330 participants into the trial over an 18 month period from 6 general practices. Follow-up is targeted for completion by January 2015 and analysis will follow.

## Discussion

The SWAP trial is investigating the clinical and cost-effectiveness of the addition of a vocational advice service to best current primary care to provide a structured approach to managing work related issues in primary care patients with musculoskeletal pain who are absent from work or struggling to remain in work. Given that early intervention is advocated, that musculoskeletal conditions are a common cause of work absence and that the majority of individuals in the UK seek their healthcare initially from their GP, we have developed and are testing a service located in primary care to address the issues of health and work early on in patients with musculoskeletal conditions. This is the first such trial in the UK. The results will provide evidence to inform primary care practice and may guide the development of services to provide support for musculoskeletal pain patients with work-related issues.

The main strength is the cluster randomised controlled trial design. The primary outcome is self-reported number of days off work over 4 months. A range of secondary outcomes will also be assessed and qualitative interviews will explore the value of a vocational advice service to GPs and NPs, patients and vocational advisors.

## Abbreviations

GP: General practitioner; ICC: Intracluster correlation coefficient; IQR: Interquartile range; NIHR: National Institute for Health Research; NHS: National Health Service; NP: Nurse practitioner; RCT: Randomised controlled trial; RTW: Return to work; SD: Standard deviation; SME: Small and medium enterprise; SWAP: Study of work and pain; UK: United Kingdom; VA: Vocational advisor.

## Competing interests

The authors declare that they have no competing interests.

## Authors’ contributions

NF, EH, CM, DvdW, ML and GW-J conceptualised and designed the study and secured funding. AB, GW-J, ML, SJ and NF wrote the full protocol. AB wrote the first draft of this manuscript. SL is the study coordinator and contributed to the operational aspects of the trial in the protocol. CM offered specific expertise in occupational health, contributed to the initial funding proposal, and training/mentoring the VAs in secondary prevention. GS conceptualised, designed and delivered the vocational advisors’ intervention, training and mentoring. KB contributed to the conceptual development of the best current care and vocational advice interventions and contributed these to the protocol. ML is the study statistician, advised on the sample size, advised on the data collection and formulated the analysis plan. SJ advised on the data collection and formulated the analysis plan for the economic evaluation. TS conceptualised and designed the qualitative study and contributed to the protocol. All authors contributed to revisions of this manuscript, have read and approved the final manuscript and take public responsibility for its content.

## Pre-publication history

The pre-publication history for this paper can be accessed here:

http://www.biomedcentral.com/1471-2474/15/232/prepub
